# Conversive and Factitious disorders: Differential diagnosis based on a case report

**DOI:** 10.1192/j.eurpsy.2024.920

**Published:** 2024-08-27

**Authors:** M. Fernandez Lozano, B. Rodriguez Rodriguez, N. Navarro Barriga, M. J. Mateos Sexmero, C. Alario Ruiz, L. Rodriguez Andrés, G. Medina Ojeda, T. Jimenez Aparicio, C. Vallecillo Adame, C. De Andres Lobo, M. A. Andreo Vidal, P. Martínez Gimeno, M. Calvo Valcarcel, M. P. Pando Fernández, L. Rojas Vazquez, M. Rios Vaquero, G. Lorenzo Chapatte, A. Monllor Lazarraga

**Affiliations:** Hospital Clínico Universitario de Valladolid, Valladolid, Spain

## Abstract

**Introduction:**

Conversive disorder is characterised by the presence of one or more involuntary neurological symptoms that are not due to a clear medical pathology. On the other hand, consciously simulated illnesses fall into two diagnostic categories: factitious disorders and malingering, which are differentiated by both the motivation for the behaviour and the awareness of that motivation. Factitious disorder behaviours are motivated by an unconscious need to assume the sick role, whereas malingering behaviours are consciously driven to achieve external secondary gains.

**Objectives:**

Study of the differences between conversion disorder and factitious disorder and their repercussions from a case of difficult diagnosis.

**Methods:**

Bibliographic review of scientific literature based on a relevant clinical case.

**Results:**

We present the case of a 14-year-old male patient. Adoptive parents. Studying in high school. Social difficulties since childhood. He comes to the emergency department on several occasions referring stereotyped movements and motor tics in the four extremities with left cervical lateralization. Increase of these symptoms in the last month, so it was decided to admit him to the pediatric hospital. After observation and study of the patient’s movements with normal complementary tests he should return home. The following day he returned to the emergency department after an episode of dizziness, mutism and emotional block. It was decided to admit him to Psychiatry for behavioral observation and differential diagnosis.

**Conclusions:**

In the assessment of patients it is essential to make an appropriate diagnosis taking into account the patient’s symptomatology and the patient’s background and life context. Conversion disorder is the unintentional production of neurological symptom, whereas malingering and factitious disorder represent the voluntary production of symptoms with internal or external incentives. They have a close history and this has been frequently confounded. Practitioners are often confronted to medically unexplained symptoms; they represent almost 30% of neurologist’s consultation. The first challenge is to detect them, and recent studies have confirmed the importance of “positive” clinical bedside signs based on incoherence and discordance. Multidisciplinary therapy is recommended with behavioral cognitive therapy, antidepressant to treat frequent comorbid anxiety or depression, and physiotherapy. Factitious disorder and malingering should be clearly delineated from conversion disorder. Factitious disorder should be considered as a mental illness and more research on its physiopathology and treatment is needed, when malingering is a non-medical condition encountered in medico-legal cases.

**Disclosure of Interest:**

None Declared

